# Editorial

**Published:** 1846-10

**Authors:** 


### THE MEDICAL EXAMINER.

PHILADELPHIA, OCTOBER, 1846.

### DR. FENNER'S LETTERS FROM THE NORTH.

Dr. Fenner, one of the editors of the "New Orleans Medical and Surgical Journal," has published in the last (September) number of that excellent periodical, a series of interesting letters on 'medical affairs' as they presented themselves to him in a recent tour to the Northern cities. These letters are exceedingly creditable to their author as a physician and a gentleman. The observations are generally judicious, and the facts rarely misunderstood; and withal there is a tone of liberal feeling pervading them which cannot be mistaken. On one point, however, Dr. Fenner has been misinformed, or has misunderstood his informant. Speaking of the Medical Clubs that exist in Philadelphia he says—"These economical clubs have gradually supplanted the ancient Wistar parties, which I understand have been nearly abandoned on account of the extravagance they fell into."

At no one time have those Wistar parties been better maintained than at present. During the last winter, the number of members was twenty-six, which, as only one entertainment is given in the week, is sufficient for half the year. The Wistar party is not, however, composed wholly or chiefly of members of the medical profession. The two fundamental laws of its existence are—*first*, that no one is eligible to membership who is not a member of the American Philosophical Society; and *secondly*, that unanimity is necessary to a choice. During the winter of 1845–6, the proportion of physicians was as eleven to twenty-six.

So much good has resulted from this Association in cultivating the kindlier sympathies, and in affording opportunities for social intercourse to scientific and literary residents and strangers, that it is not likely to suffer decadency, and will probably be continued long after the present generation has passed away. The main regulations are—that the member at whose house the party is given, may invite citizens, but it is recommended, that the number shall not exceed forty:—that all the members may introduce strangers; and that "the entertainment shall consist only of a collation of a single course; and it is recommended that it be frugal." The "Wistar Association," essentially in its present form, commenced at the death of Dr. Wistar, who had been in the habit of holding weekly re-unions. It has, consequently, already existed without any interruption for thirty-six years.

---

### ANNUAL CIRCULARS OF MEDICAL COLLEGES.

In addition to those announced in our last number, we have received the following Annual Circulars, viz.:

"*Report of the Medical Department of the University of Pennsylvania, for the year* 1846. *To the Alumni of the School. By the Medical Faculty.*"

The Faculty say, in this "Report," that "A comparison of the numbers who have attended the instructions, and received the honours of the Institution, during the last twelve months, with those of former years, will afford to its friends a gratifying evidence that it has lost no ground in the public estimation." The number of "Matriculants" at the last Session was - - - - - - - - 464

Of Graduates - - - - - - - - 171

The names of the gentlemen who were announced last year as Lecturers on Clinical Surgery and Clinical Medicine, as well as the name of the Demonstrator of Anatomy, are omitted in this publication. Whether any new arrangements have occasioned this change is not stated.

"*Annual Announcement of Jefferson Medical College of Philadelphia. Session of* 1846-7."

This "*Announcement*" contains a full account of the number of Students and Graduates of the last year; a sketch of the courses of instruction given by the different Professors; number of patients prescribed for and operated on during the past year in presence of the Class, and other matters of general interest. We derive from it the following facts, which cannot fail to gratify all who feel an interest in the growing prosperity of the Institution:—

| | | | | |
|---|---|---|---|---|
| "In the Session | 1840—41 | the number of | Students was | 163 |
| " | 1841—42 | " | " | 209 |
| " | 1842—43 | " | " | 229 |
| " | 1843—44 | " | " | 341 |
| " | 1844—45 | " | " | 409 |
| " | 1845—46 | " | " | 469 |

| | | | |
|---|---|---|---|
| The number of | Graduates in | 1842—43 was | 47 |
| " | " | 1843—44 " | 117 |
| " | " | 1844—45 " | 116 |
| " | " | 1845—46 " | 170 |

Thus it appears that the number in attendance on the lectures at this Institution the last Session was the largest in the country.

The new College edifice, a neat representation of which is on the title page of the Announcement, is now completed.

"*Annual Circular of the Massachusetts Medical College, with a History of the Medical Department of Harvard University, a Catalogue of the Graduates, &c.* Boston: 1846."

The brief history contained in this neatly got up pamphlet of the Medical School of Boston and the Massachusetts General Hospital, is exceedingly interesting, and, in some measure, it may also be regarded as a biographical sketch of the principal Boston Physicians and Surgeons. We have been very much struck with the information contained in a note in relation to the Hospital, from which it appears that the donations which the Institution has received, in sums of $5,000 and over, amount to $394,000! One bequest alone was of $119,000, and another $89,000! Can any other city in America boast of such munificence?

The increasing prosperity of the School has rendered it necessary for the Faculty to provide a new college building of ample dimensions. "The expense of this building has been provided for, partly by the sale of the old College and land, partly by a liberal donation of an ample lot for the reception of the new College by George Parkman, M. D., of Boston, and partly by voluntary contributions of other wealthy citizens of our metropolis, whose aid is rarely sought in vain for the promotion of necessary scientific or charitable objects." So says the circular. When will Philadelphia do as much for her Medical Colleges? Here, every dollar comes from the pockets of the professors.

The Class attending the last course of lectures in the Boston Medical College was composed of 159 students—a greater number than in any previous year.

"*Annual Announcement of the Medical Department of the St. Louis University. Session of* 1846—7."

The Class of the last Session numbered 52, which, it is stated, is an increase over the preceding year. Twelve gentlemen were graduated at the commencement in March.

Henry M. Bullitt, M. D., of Louisville, Kentucky, has been appointed to the Chair of Physiology and Pathology, vacated by the resignation of Professor Hall. Professor Bullitt is an able and accomplished physician, and will do honour to the station to which he has been elected.

"*Annual Circular of the Medical Department of Illinois College. Jacksonville, Ill., July* 1*st*, 1846."

The Faculty of this Institution consists of six Professors. The Lectures commence annually on the first Monday of November, and continue four calendar months. Graduates of Session 1845—6, fourteen. Number of the Class not stated.

"*Catalogue of the Philadelphia School of Anatomy,* 1845—6, *and Announcement for* 1846—7. *By James McClintock, M. D.*"

Number of the Classes during the Season, 165.

This is one of the private schools for giving instruction in practical Anatomy that exist in Philadelphia, and of which there are several. The success which attends them, notwithstanding the extensive means of the same kind afforded by the Colleges, shows the great attention paid to this subject in Philadelphia, and that there is no great lack of *materiel*, which, from certain publications that annually appear, the public might suppose is only to be had in a single city in the Union!

---

MEDICAL SCHOOL AT BUFFALO, N. Y.

From a notice in the last number of the Buffalo Medical Journal, we perceive that another Medical College has been organized, to be located in the thriving town of Buffalo, in the State of New York. The faculty is constituted as follows:—

Chemistry and Pharmacy, James Hadley, M. D.

Physiology and Medical Jurisprudence, Charles B. Coventry, M.D.

General and Special Anatomy, James Webster, M. D.

Pathology and Materia Medica, Charles A. Lee, M. D.

Principles and Practice of Surgery and Clinical Surgery, Frank H. Hamilton, M. D.

Obstetrics and Diseases of Women and Children, James P. White, M. D.

Principles and Practice of Medicine and Clinical Medicine, Austin Flint, M. D.

Of these seven Professors, the first five named are also professors in Geneva Medical College, and continue their connection with the latter Institution. For the convenience of the parties, the lectures are to be given at Geneva in the winter, and at Buffalo in the spring, the *first course.* Altogether, the scheme looks to us like a *transfer* of the Medical College of Geneva to Buffalo, the latter having the larger population and being in many respects the more eligible place, and we apprehend that to this complexion it will come at last.

**Statistics of Medical Colleges.**—Some time since, we published an incomplete list of the Students and Graduates at the different Medical Schools throughout the country, and invited its correction by other journalists who might be cognizant of the facts. We now copy from the Buffalo Medical Journal a list in which most of the deficiencies in our own are supplied. In our contemporary, however, we have discovered some material errors which we have corrected. Thus, the Class of Jefferson Medical College is set down in that Journal as 459 instead of 469; University of Pennsylvania 471, instead of 464; Missouri University, St. Louis, the graduates are stated to be 29, instead of 12; and the total number of graduates is greater than our computaiton. It is likely that the account is not yet quite correct, but it is as nearly so as we can make it with the data we possess.

| | Class 1845-6. | Graduates. |
|---|---|---|
| University of Pennsylvania, - - - - - | 464 | 171 |
| Jefferson Medical College, Philadelphia, - - | 469 | 170 |
| University of City of New York, - - - | 425 | 131 |
| College of Physicians and Surgeons, N. Y., - | 200 | 38 |
| Geneva Medical College, N. Y., - - - | 178 | 39 |
| Albany Medical College, N. Y., - - - | 115 | 42 |
| Harvard University, Boston, Mass., - - | 159 | 31 |
| Berkshire Medical Institution, Mass., - - | 142 | 35 |
| Castleton Medical College, - - - - | 140 | 36 |
| Yale Medical College, New Haven, Conn., - | 53 | 19 |
| Cleaveland Medical College, Ohio, - - | 160 | 52 |
| Willoughby Medical College, Ohio, - - | 164 | 40 |
| Vermont Medical College, Woodstock, - | | 24 |
| Ohio Medical College, Cincinnati, - - - | 195 | 46 |
| Transylvania University, Lexington, Ky., - | 171 | 64 |
| Louisville Medical Institute, Ky., - - | 345 | 43 |
| University of Maryland, Baltimore, - - | 147 | 40 |
| Bowdoin Medical College, Brunswick, Me., - | 73 | 19 |
| Rush Medical College, Chicago, Ill., - - | 50 | 9 |
| Indiana Medical College, La Porte, - - | 81 | 18 |
| Medical College of Louisiana, New Orleans, - | 103 | 20 |
| Medical College of Georgia, Augusta, - - | 112 | 15 |
| Missouri University, St. Louis, - - - - | 53 | 12 |
| Kemper College, St. Louis, - - - | | 11 |
| Total, | 3,999 | 1,125 |

---

DEATH OF DOCTOR BOSTOCK.

From the London Lancet of the 22d of August, we learn that Dr. Bostock, author of "Elements of Physiology," and various treatises on Medical subjects, died recently of an attack of cholera, at the age of seventy-three. Dr. B. was a native of Liverpool, but removed to London in 1817. He was the associate of Davy, Wollaston, Young, and other distinguished philosophers of his day, and himself occupied many situations allotted only to those of distinguished merit. "In private life, no man was more respected and beloved. He was equally ready to impart the overflowing of his sensitive and affectionate heart, and his varied stores of knowledge with which his intelligent mind abounded."

